# Determination of short-chain chlorinated paraffins in multiple matrices of Arctic using gas chromatography-electron capture negative ion-low resolution mass spectrometry

**DOI:** 10.1016/j.mex.2018.07.017

**Published:** 2018-07-24

**Authors:** Huijuan Li, Jianjie Fu, Wenxiao Pan, Pu Wang, Yingming Li, Qinghua Zhang, Yawei Wang, Aiqian Zhang, Yong Liang, Guibin Jiang

**Affiliations:** a Key Laboratory for Applied Technology of Sophisticated Analytic Instrument, Qilu University of Technology (Shandong Academy of Science), Jinan, China; b State Key Laboratory of Environmental Chemistry and Ecotoxicology, Research Center for Eco-Environmental Sciences, Chinese Academy of Sciences, Beijing, 100085, China; c College of Resources and Environment, University of Chinese Academy of Sciences, Beijing, 100049, China; d Institute of Environment and Health, Jianghan University, Wuhan, 430056, China

**Keywords:** Gas Chromatography-Electron Capture Negative Ion-low resolution Mass Spectrometry (GC-ECNI-LRMS), Accelerated solvent extraction (ASE), Multilayer silica-florisil column, GC-ECNI-MS

## Abstract

Gas chromatography-electron capture negative ion-low resolution mass spectrometry (GC-ECNI-MS) was used for the quantification of short-chain chlorinated paraffins (SCCPs) in multiple matrices of Arctic. Samples were spiked with surrogate standards (^13^C_10_-*trans*-chlordane) and extracted with dichloromethane and hexane (1:1, *v/v*) using accelerated solvent extraction (ASE). The extract was cleaned using a multilayer Silica-Florisil column and ε-HCH was added before instrument analysis. The SCCPs were analyzed using a gas chromatograph (GC) in electron capture negative ion (ECNI) mode coupled with a 7000B triple quadruple mass spectrometer (MS) in single quad mode. The calibration is performed using three commercial standards (chlorine contents of 51.5%, 55.5%, and 63.0%). A reasonable linear correlation for commercial standards was found between chlorine content and total response factor (*R^2^* = 0.96). To ensure instrument sensitivity, the SCCP congeners were divided into four groups by the optimized combinations (C_10_, C_11_, C_12_ and C_13_) and subjected to analysis by four individual injections. This method is suited to analyse the total concentration of SCCPs and individual SCCP congener groups in the environmental analysis.

• Accelerated solvent extraction offers a lower cost of time for per sample and reducing solvent consumption.

• The presented quantification procedure makes the quantification of SCCPs independent from the chlorine content of the used standard mixtures.

• The samples were subjected to analysis by four individual injections for the instrument sensitivity, and the total concentration of SCCPs and CP congener groups can be obtained at the same time.

## Graphical abstract

**Figure:**
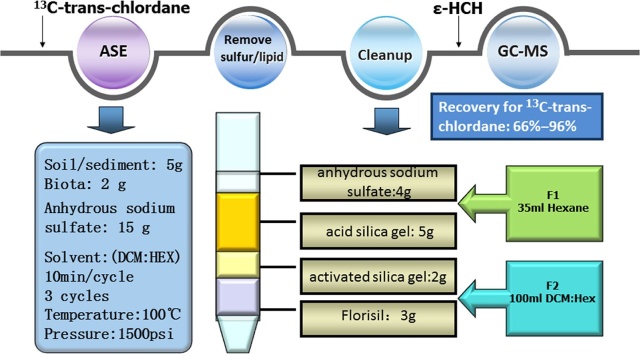

**Specification Table**

**Table:** 

Subject Area	*Environmental Science*
More specific subject area:	*Environmental analytical chemistry*
Method name:	*Gas Chromatography-Electron Capture Negative Ion-low resolution Mass Spectrometry (GC-ECNI-LRMS)*
Name and reference of original method	New quantification procedure for the analysis of chlorinated paraffins using electron capture negative ionization mass spectrometry
Resource availability	*Accelerated Solvent Extraction (Dionex ASE 350)7890?A GC - 7000B MS (Agilent, USA)*

## Method details

### Chemicals

**Fig. 1:**
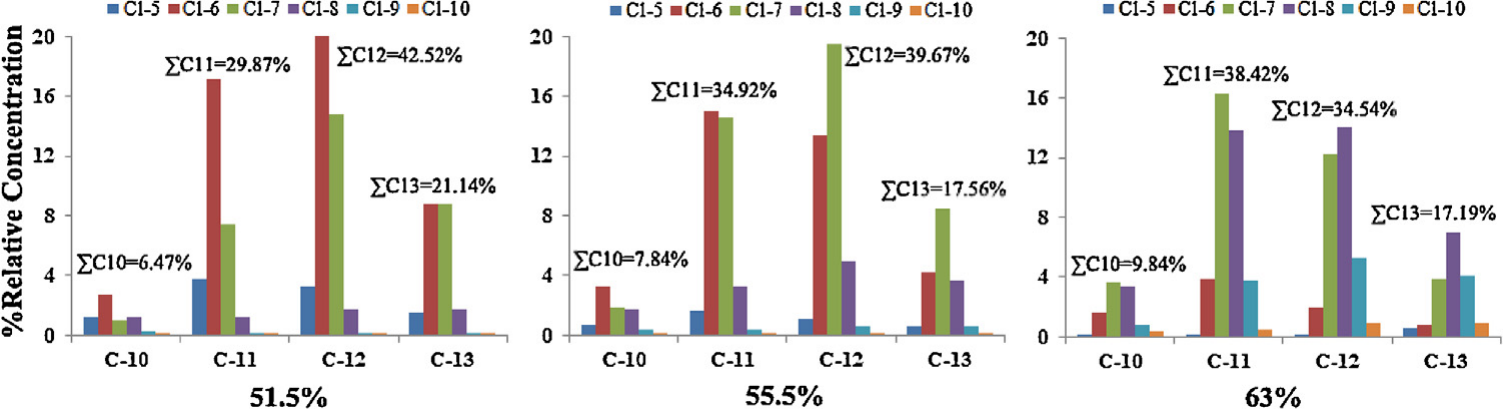
Congener group pattern for commercial references of SCCPs (C_10-13_, 51.5%, 55.5% and 63.0% chlorine content).

The commercial standards of the SCCPs (chlorine contents of 51.5%, 55.5%, and 63.0%, with the congener group patterns shown in Fig. 1) at concentrations of 100 ng/μL in cyclohexane and ε-hexachlorocyclohexane (*ε*-HCH, solution in cyclohexane, 10 ng/μL) were purchased from Ehrenstorfer GmbH (Augsburg, Germany). The ^13^C_10_-*trans*-chlordane (100 ng/μL solution in *n*-nonane, purity 99%) was purchased from Cambridge Isotope Laboratories (Andover, USA). Pesticide-grade dichloromethane, *n*-hexane, acetone, cyclohexane, and toluene were purchased from JT Baker (Phillipsburg, NJ). Florisil (60–100 mesh) and silica gel (180–280 mesh) were purchased from Merck (Whitehouse Station, NJ). Anhydrous sodium sulfate was purchased from Sinopharm Chemical Reagent Beijing Co., Ltd.

Silica gel was activated at 550 °C for 12 h, Florisil was activated at 140 °C for 7 h, and anhydrous sodium sulfate was activated at 660 °C for 6 h before use. A mixture of 30% (40%) acid silica gel was prepared by mixing 100 g of activated silica gel and 44 g (66 g) of concentrated sulfuric acid.

### Sample pre-treatment

Approximately 5 g of soil/sediment samples and 2 g of biota samples were spiked with surrogate standard (1 ng of ^13^C_10_-*trans*-chlordane), mixed with 15 g of anhydrous sodium sulfate and extracted with dichloromethane and hexane (1:1, *v/v*) using accelerated solvent extraction (Dionex ASE 350). The cell was heated at 100 °C and reached a pressure of 1500 psi. The thermal equilibration time was 10 min, and the static extractions were performed within three cycles, with the cell purged with gaseous nitrogen for 120 s. For biota, the extraction was rotary-evaporated to dryness to determine the lipid content and was later re-dissolved in 60 mL of hexane and dichloromethane (1:1). Activated copper granules were added to the soil/sediment extraction to remove elemental sulfur; then, 40% acid silica gel (*w/w*) was added to the biological extraction to remove lipids and other types of interference and filtered through approximately 5 g of anhydrous sodium sulfate. The cleanup procedure for the SCCPs was based on our previously reported methods [1]. In detail, the extraction was rotary-evaporated to approximately 2 mL and then cleaned and fractionated on a multilayer Silica-Florisil composite column that consisted of 3 g of Florisil, 2 g of activated silica gel, 5 g of acid silica gel (30%, *w/w*) and 4 g of anhydrous sodium sulfate from the bottom to the top. The column was pre-cleaned with 50 mL of hexane, and the extract was eluted in sequence with 40 mL of hexane (first fraction) and 100 mL of dichloromethane/hexane (1:1, *v/v*) (second fraction). The second fraction was concentrated, and the solvent was exchanged to cyclohexane to a final volume of 100 μL. Finally, 10 ng internal standard of *ε*-HCH was added before instrument analysis.

## Instrument analysis

Instrumental analysis of the SCCPs was performed on a 7890 A gas chromatograph (GC) in electron capture negative ion (ECNI) mode coupled with a 7000B triple quadruple mass spectrometer in single quad mode (Agilent, USA). The extract (1 μL) was injected using a 7683B Series Injector (Agilent, USA) in splitless mode into a DB-5MS capillary column (30-m length, 0.25-mm i.d., 0.25-μm film thickness; Agilent, CA) at an injector temperature of 275 °C. Helium was used as the carrier gas at a flow rate of 1.0 mL/min. The oven temperature program was as follows: 1 min isothermal at 100 °C, increased to 160 °C at a rate of 30 °C/min, maintained for 5 min, and then increased to 310 °C at 30 °C/min and maintained for 17 min A low-resolution mass spectrometer was employed in the ECNI mode with methane as the reagent gas. The transfer line and ion source temperatures were set to 275 °C and 200 °C, respectively. The most abundant isotope was used for quantification, and the second most abundant isotope was used for identification. To ensure instrument sensitivity, the SCCP congeners were divided into four groups by the optimized combinations (C_10_, C_11_, C_12_ and C_13_) and subjected to analysis by four individual injections.

## Identification and quantification

**Fig. 2:**
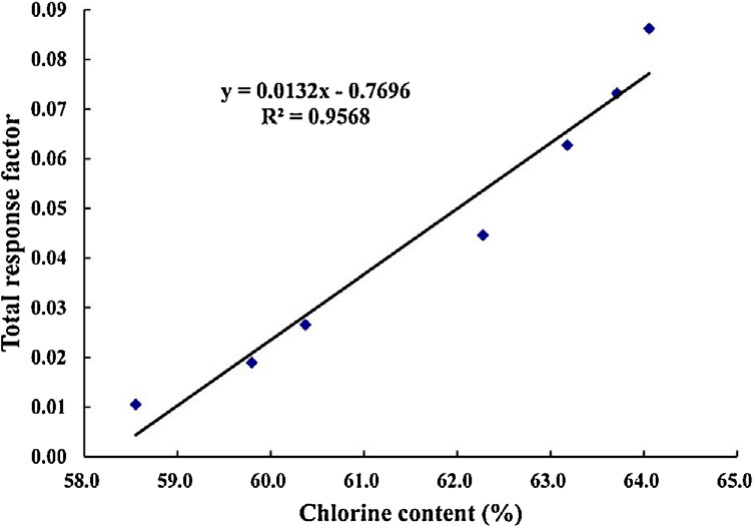
Correlation between chlorine content and total response factor for commercial standards.

Identification of the CP congener groups was performed by comparisons of the retention time, signal shape, and correct isotope ratio, according to Reth et al. [2]. The actual relative integrated signals for each congener were obtained by correcting the SIM signals of the [M-Cl]^−^ ions from the isotopic abundance and response factors. Congener group abundance profiles were established using the actual relative integrated signals, followed by a chemical calculation to determine the relative concentrations of the molecular component in the commercial standards and environmental samples. Based on this method, the quantification is reliable even when the degree of chlorination between the samples and the commercial standards is different. Three commercial standards of the SCCPs (chlorine contents of 51.5%, 55.5%, and 63.0%) were purchased from Ehrenstorfer GmbH. Additionally, a solution of 53% was prepared from the SCCP mixtures with 51.5% and 55.5% (1:1, *V/V*) as well as from the SCCP mixtures with 55.5 and 63% (59.5%), 55.5 and 59.5% (57%), 59.5 and 63% (61%). Seven SCCP mixtures of different chlorine content (51.5%–63%) were tested by GC-ECNI-MS. A reasonable linear correlation for the commercial standards was found between chlorine content and total response factor (Fig. 2, *R*^2^ = 0.96).

## Quality assurance and quality control (QA/QC)

**Fig. 3:**
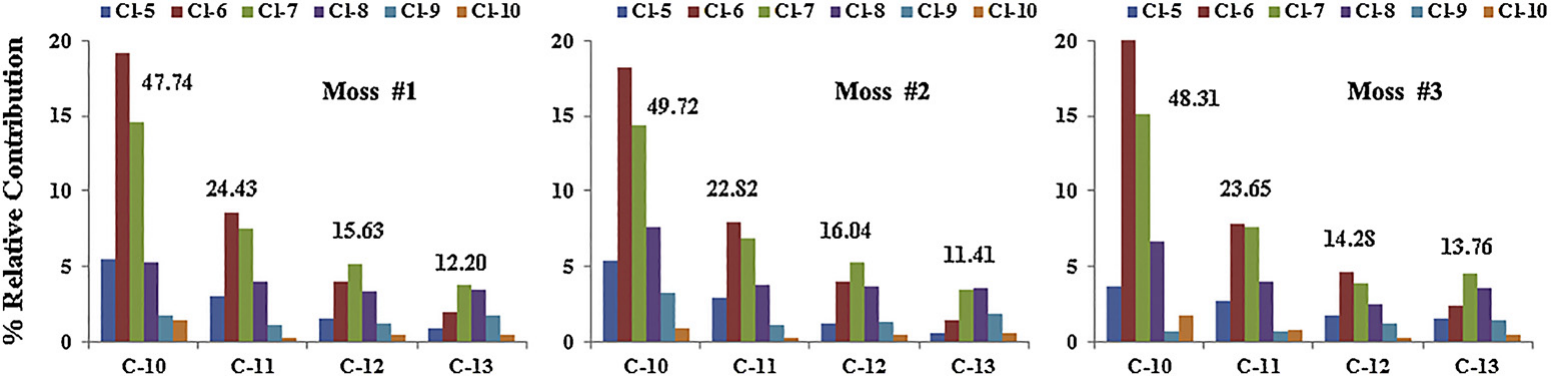
Congener group profiles of SCCPs in Arctic moss sample.

To ensure the validity of the analytical identifications and quantifications, strict quality assurance and control measures were employed. Sample equipment was pre-washed with hexane and dichloromethane before transport to the Arctic. All samples were dried and homogenized immediately at the Chinese Arctic Yellow River Station, packed in aluminium foil and sealed in clean plastic bags. After transport to the laboratory in Beijing, China, the samples were stored at −20 °C in a specialized refrigerator for polar samples. Anhydrous sodium sulfate was used as a blank control throughout the treatment process (sampling, transport and storage, sample drying, homogenizing, extraction, clean-up, and instrumental analysis). The concentrations of SCCPs detected in anhydrous sodium sulfate were 0.56–2.17 ng/g, which were less than 5% of the average sample concentrations (165.2 ng/g dw). The accuracy and the stability of the analysis results were validated by Arctic moss samples. An Arctic moss sample with sufficient amount was analysed for three times. The SCCPs concentrations in moss samples were 232.4 ng/g dw, 289.1 ng/g dw, and 268.6 ng/g dw, respectively, and relative contributions of SCCP congener groups showed the similar profiles (Fig. 3). Moreover, singular value appearing in the experience was validated by duplicate measurements in the present study.

All of the sample treatment steps were conducted in a laboratory fume hood (American T.O.F. International Science-Lab Co., Ltd.). Glassware was soaked in deionized water with a neutral wash solution of Decon 90 for over 6 h and then washed at least six times with tap water and three times with deionized water. Finally, glassware was heated to 450 °C in a muffle furnace and thoroughly rinsed twice with dichloromethane before use. Procedural blanks were processed for each batch of samples, to control for contamination throughout extraction, clean-up, and instrumental analysis. In general, the levels of SCCPs in the procedural blanks were close to or under the limit of detection (LOD), which was estimated as 0.1 ng. The recovery of ^13^C_10_-*trans*-chlordane was between 66% and 96%. The spiked recoveries for the three SCCP references with chlorine contents of 51.5%, 55.5%, and 63.0% were between 81% and 116%.
